# Investigation of the Solubility of Elemental Sulfur (S) in Sulfur-Containing Natural Gas with Machine Learning Methods

**DOI:** 10.3390/ijerph20065059

**Published:** 2023-03-13

**Authors:** Yuchen Wang, Zhengshan Luo, Jihao Luo, Yiqiong Gao, Yulei Kong, Qingqing Wang

**Affiliations:** 1 School of Management, Xi’an University of Architecture and Technology, Xi’an 710055, China; 2 School of Computer Science, Beijing Institute of Technology, Beijing 100081, China

**Keywords:** sulfur-containing natural gas, sulfur solubility, machine learning, hydrogen sulfide

## Abstract

Some natural gases are toxic because they contain hydrogen sulfide (H_2_S). The solubility pattern of elemental sulfur (S) in toxic natural gas needs to be studied for environmental protection and life safety. Some methods (e.g., experiments) may pose safety risks. Measuring sulfur solubility using a machine learning (ML) method is fast and accurate. Considering the limited experimental data on sulfur solubility, this study used consensus nested cross-validation (cnCV) to obtain more information. The global search capability and learning efficiency of random forest (RF) and weighted least squares support vector machine (WLSSVM) models were enhanced via a whale optimization–genetic algorithm (WOA-GA). Hence, the WOA-GA-RF and WOA-GA-WLSSVM models were developed to accurately predict the solubility of sulfur and reveal its variation pattern. WOA-GA-RF outperformed six other similar models (e.g., RF model) and six other published studies (e.g., the model designed by Roberts et al.). Using the generic positional oligomer importance matrix (gPOIM), this study visualized the contribution of variables affecting sulfur solubility. The results show that temperature, pressure, and H_2_S content all have positive effects on sulfur solubility. Sulfur solubility significantly increases when the H_2_S content exceeds 10%, and other conditions (temperature, pressure) remain the same.

## 1. Introduction

**Figure 1:**
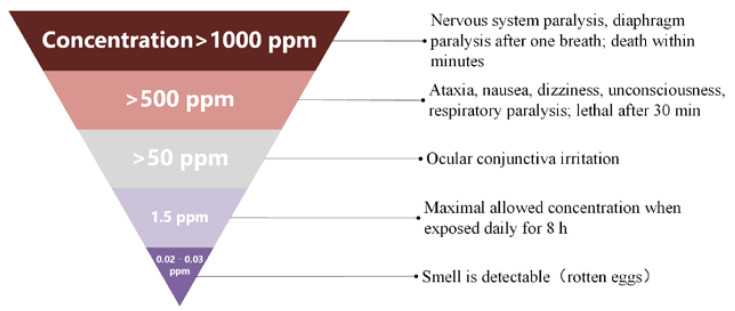
Effects of different concentrations of H_2_S on humans.

The presence of sulfur (S) in the environment is the result of natural processes and human activities [1,2]. Sour gas fields contain large amounts of elemental sulfur, and the solubility of sulfur varies under different external conditions [3]. Changes in sulfur solubility are accompanied by the generation of different products: $H_{2}S+S_{x}↔H_{2}S_{x+1}.$ As the equilibrium moves toward the production of polyhydrogen sulfide, the solubility of elemental sulfur in natural gas increases, and the amount of H_2_S decreases. The reaction proceeds in the reverse direction: sulfur solubility decreases and promotes the production of H_2_S. Based on the dissolution pattern of sulfur, researchers can control the production of H_2_S. H_2_S is a highly toxic gas that can negatively impact the environment and biosecurity [4]. For example, it can acidify the soil, freshwater, and marine ecosystems, making forests more susceptible to frost, drought, and insect infestation. The threat of H_2_S to human health is illustrated in Figure 1 [5]. The environmental authorities place severe restrictions on the gas’s sulfur concentration when using natural gas that contains sulfur [6,7]. The deep desulphurization of sour gas is important to meet production objectives and emission standards. However, the current desulfurization process consumes a significant amount of energy, making it impossible to rigorously fulfill emission standards (e.g., “Emission Standard of Air Pollutants for Onshore Oil and Gas Exploitation and Production Industry”: GB 39728—2020), while also failing to meet the criteria for sustainable development [8]. These outcomes could be the result of improper operating circumstances that do not appropriately match the sulfur dissolving pattern of a feed gas, which leads to a low desulfurization efficiency. In conclusion, considering the risks of sulfur-containing natural gas to human health, as well as its value to the environment and energy sustainability, researchers need to understand the changing patterns of sulfur solubility and make accurate predictions of sulfur solubility [9,10].

**Figure 2:**
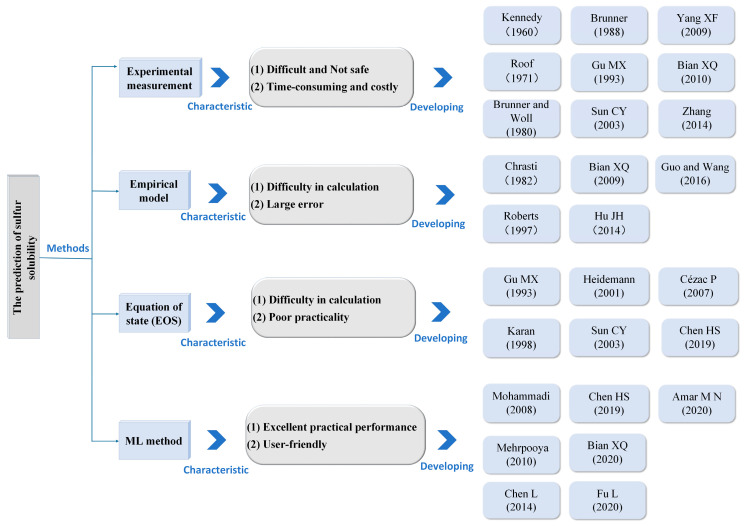
Methods for obtaining sulfur solubility and developments.

Sulfur solubility can be obtained using four different methods, as shown in Figure 2. Experimental methods are the most accurate and reliable means of determining sulfur solubility. However, the cost of experimentation is high and there may be safety risks [11]. An equation of state (EOS) and empirical models are limited by high computational requirements. Furthermore, these methods are not generalizable and are limited to specific systems [12]. Since 2008, machine learning (ML) methods, which have the advantages of a fast response time and generalization, have been increasingly used to predict sulfur solubility. Although weighted least squares support vector machine (WLSSVM) and random forest (RF) models have proved advantageous for predicting a variety of problems, the application of these two models for predicting sulfur solubility is rarely reported in the literature. Various ML methods are compared in Table 1 [9,13,14,15,16].

To obtain a robust and efficient ML model for predicting sulfur solubility, researchers need to fine-tune the hyperparameters that define the ML model architecture, such as the penalty parameter in support vector machines. By carefully selecting the ideal hyperparameters, hyperparameter optimization (HPO) aims to construct the best ML model [17]. According to Table 1, it can be concluded that researchers typically employ a single, intelligent algorithm to choose the parameters for the ML model. The no-free-lunch (NFL) theorem contends that each approach has advantages and disadvantages [18]. With integrated learning techniques, multiple algorithms or models are cleverly integrated to improve prediction accuracy [19,20]. A meta-heuristic algorithm may be a better choice for HPO problems in some tasks because it is effective at a range of tasks and can find the best solution [21]. Although the metaheuristic algorithm has many benefits, it still carries a risk of entering local optima and cannot ensure the detection of global optima. Utilizing the integration idea, the whale optimization algorithm (WOA) and genetic algorithm (GA) are combined using a serial technique to inhibit the algorithm from entering the local optimum and to enhance its global search capability [22,23]. To increase algorithm diversity, the crossover operator (cOPR) and variation operator (vOPR) of the GA are incorporated into the WOA. Additionally, the adaptive weight update strategy (awuST) is used to accelerate convergence and enhance convergence accuracy. Convergence is accelerated, and convergence accuracy is enhanced by the cross-variance operator (c-vOPR), together with the awuST.

When building machine learning models, the vast majority of models require an adequate number of samples to be built. However, in many research contexts, the sample size is not sufficient, and the study of sulfur solubility is one of them. Small sample sizes make it difficult to train ML models. Cross-validation (CV) can assist researchers in extracting more information from sparse data. Nested cross-validation (nCV) is an enhanced version of CV that can effectively help train small samples to obtain the best machine learning model [24]. However, the standard nCV not only requires extensive calculations, but may also select too many irrelevant features, thus affecting the interpretation of the model. The consensus nested cross-validation (cnCV), which was recently developed, successfully resolves these issues [25]. In this study, cnCV was used to assist the training of the sulfur solubility prediction model.

Although ML models have been used to achieve sulfur solubility, they still have some shortcomings:

(1) Researchers appear not to have focused on the impact of hyperparameter optimization in ML models for sulfur solubility prediction on model performance. Moreover, most of the studies typically employ a single algorithm to build ML models. Single algorithms usually have unavoidable drawbacks that may degrade the models’ capabilities.
(2) Despite the limited actual sample of sulfur solubility, researchers have not focused on its limitations in training ML models or the use of WLSSVM and RF for predicting sulfur solubility despite their efficiency and promise.
(3) In previous studies, scholars did not take remedial measures against the black-box characteristics of the ML model. The lack of interpretability of experimental results may limit scholars’ exploration of sulfur solubility variation patterns in practical applications.

Combining the above studies, the proposed two integrated optimization machine learning models, WOA-GA-WLSSVM and WOA-GA-RF, can form a good solution to the shortcomings of previous ML models for sulfur solubility prediction. The main contributions of the study are summarized below.

(1) For hyperparameter optimization, with the help of c-vOPR and awuST, the new method of a whale optimization–genetic algorithm (WOA-GA) balances accuracy with efficiency, while improving global search capabilities and reducing the risk of slipping into local extremes in the hyperparameter search process.
(2) The WOA-GA-WLSSVM and WOA-GA-RF integrated optimization ML models were created. To train ML models that can accurately predict sulfur solubility, this study uses cnCV as a tool to obtain sufficient information from a limited sample. The performance of the suggested models, as well as their stability and reliability, are analyzed from various angles.
(3) The generic positional oligomer importance matrix (gPOIM) is used to estimate how each variable affects sulfur solubility, from which patterns of variation in sulfur solubility are extracted.

This paper is organized as follows. In Section 2, modeling techniques are introduced, and the modeling process is explained. In Section 3, the predicted results and the stability and reliability of the model are critically assessed and validated. The analysis of the contribution of characteristics, which revealed the significance of the variables and helped to analyze the sulfur solubility pattern, is described. Section 4 presents the conclusions and recommendations of this study.

## 2. Methods

### 2.1. Optimization Methods

#### 2.1.1. Consensus Nested Cross-Validation (cnCV)

Small sample sizes make it difficult to train ML models. Cross-validation (CV) can assist researchers in extracting more information from sparse data. A CV allows all datasets to be randomly grouped and used for both training and validation. As a result, CV can be used to solve the insufficient data problem. A k-fold cross-validation (k-fold CV) is a common cross-validation method. In contrast to k-fold CV, the execution of nCV consists of two loops, i.e., an outer loop and an inner loop. As such, it is possible to avoid the leakage of information from data and, therefore, obtain relatively low biases in model scoring [26,27]. However, nCV may select some irrelevant features, which complicates the model and is economically unsustainable [25]. Table 2 lists the disadvantages of k-fold CV and nCV. cnCV was developed using the feature stability concept of differential privacy to address the shortcomings of standard nCV (as depicted in Table 2). Differential privacy derives from cryptography and is essentially a trade-off between the degree of privacy protection and data availability. It extracts useful information about variables while limiting the leakage of information. The operation of cnCV is broadly divided into two parts [25]. The inner loop performs a cross-validation to determine the optimal hyperparameters and features of the model, which are used by the outer loop. The outer loop provides training data for the inner loop, while retaining some data for testing the inner loop model.

**Figure 3:**
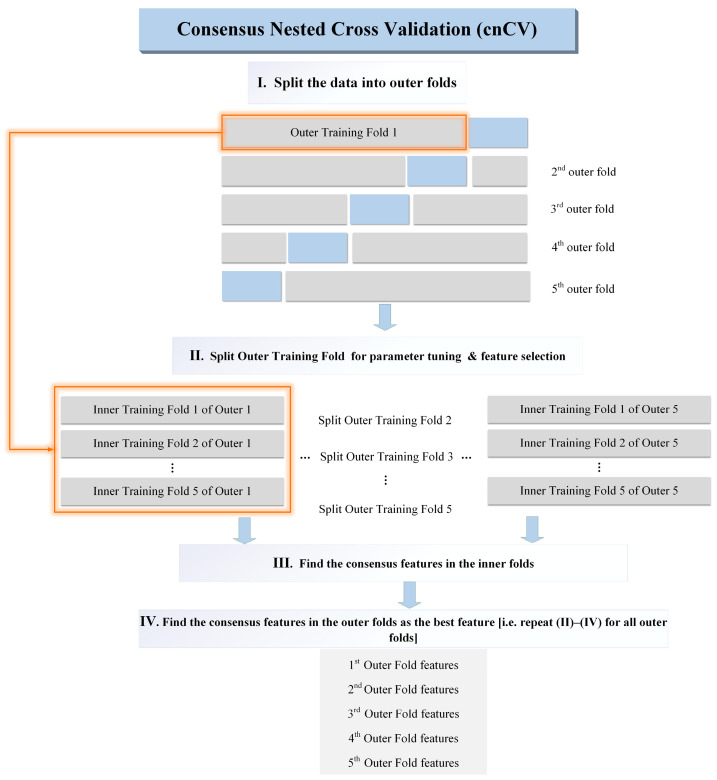
Operation diagram of cnCV.

Figure 3 explains the procedure diagram of the algorithm. Firstly, the data are divided into outer folds, and then each outer fold is split. The ReliefF algorithm was used to calculate the relief scores of each feature in the inner fold. The same features with positive relief scores in each inner fold were used as consensus features in the inner fold (for example, feature “ABC” is shared by the other inner folds; therefore, feature “ABC” is used as the consensus feature in the inner fold). The consensus features of all inner folds are used to represent the outer fold set of features. Next, consensus features are identified in the outer fold using the same method. In the end, all consensus features of the outer folds are used as the best features.

#### 2.1.2. The Hybrid Optimization Algorithm WOA-GA

Optimization is one of the core components of ML, and HPO is a necessary step in the model optimization process that is crucial to achieving an excellent performance in the ML model [24,28]. The essence of HPO is to use optimization algorithms to learn and select the optimal hyperparameters from the given data to determine the extreme value of the objective function [21,29]. In other words, the ultimate goal of HPO is to achieve objective function optimization [30].

For the HPO problem, a metaheuristic algorithm is an effective tool [29]. The GA has a good ability to find the global optimum and reduce the possibility of falling into the local optimum due to the variation operator (vOPR) and crossover operator (cOPR). However, the complex structure means that the GA will take longer to implement. The basic idea of the WOA is derived from the predatory behavior of humpback whales. Humpback whales not only contract their envelope when feeding, but also swim in a spiral pattern towards their prey; therefore, each humpback whale has a 50% probability (probability value *p* of predatory behavior) of choosing either a shrinking encircling mechanism or a spiral updating position. The group-following property causes most individuals to converge to the region of the current optimal individual. The final search result will easily fall within the local optimum if the current optimal individual is a local optimum. Incorporating GA’s cOPR and vOPR into WOA can enhance the global search capability to avoid falling into a local optimum; that is, WOA-GA is used as an optimization tool for the objective function. The core principle of the WOA-GA is shown below.

(1) Cross-variance operator (c-vOPR)

The GA takes a real-valued encoding and randomly generates a probability value *p* of predatory behavior in the range of (0, 1). If *p ≥* 0.5, the individual is mutated according to Equation (1), with a mutation probability *P_m_* of obtaining a new individual:

(1) $$X^{*}\left(t+1\right)=X^{*}\left(t\right)+P_{m}\left|X^{*}\left(t\right)-X(t)\right|$$

where *t* is the present number of iterations. In the *t*-th generation, X(t) represents the location of individual whales, and $X^{*}\left(t\right)$ is the location of the best individual.

The definition of the stochastic vector is as follows:

(2) A=2ar−a

where *r* is a stochastic vector in the range of (0, 1); a is a convergence factor with convergence bounds from 2 to 0, defined as $a=2-2t/T_{\max}$ (*T*_max_ is the maximum number of iterations).

Assuming *p* < 0.5 and |A| < 1, the individual further selects the global optimal individual with the crossover probability *P_c_* to perform the crossover operation with the current individual and obtains a new individual to replace the current individual. The crossover formula is:

(3) $$\left\{ \begin{array}{l} X_{i}\left(t+1\right)=P_{c}\times X_{i}\left(t\right)+\left(1-P_{c}\right)X_{j}\left(t\right)\\ X_{j}\left(t+1\right)=\left(1-P_{c}\right)\times X_{i}\left(t\right)+P_{c}X_{j}\left(t\right) \end{array}\right.$$

where *P_m_* and *P_c_* take random values in the range of (0, 1), $X_{i}$ and $X_{j}$ are the global optimal individual and the current individual, respectively.

(2) Adaptive weight update strategy (awuST)

Though the c-vOPR improves the performance of the algorithm to some extent, its complex structure may cause the algorithm to fail to balance the global search capability with the local search capability. Therefore, the awuST is introduced to balance the global and local search via adjusting the value of the weight *ω* [31]. The output values are updated with adaptive variants when *p* < 0.5 and *A* ≥ 1. The following formula illustrates this strategy:

(4) $$X\left(t+1\right)=\omega X^{*}\left(t\right)-A*\left|C*X^{*}\left(t\right)-X\left(t\right)\right|$$

(5) $$\omega =\omega_{\max}-G_{i}*\frac{\omega_{\max}-\omega_{\min}}{G_{\max}}$$

where *ω* is the weight factor; random vector C=2r; *G*_max_ is the maximum number of iterations; and *G_i_* is the current number of iterations.

**Figure 4:**
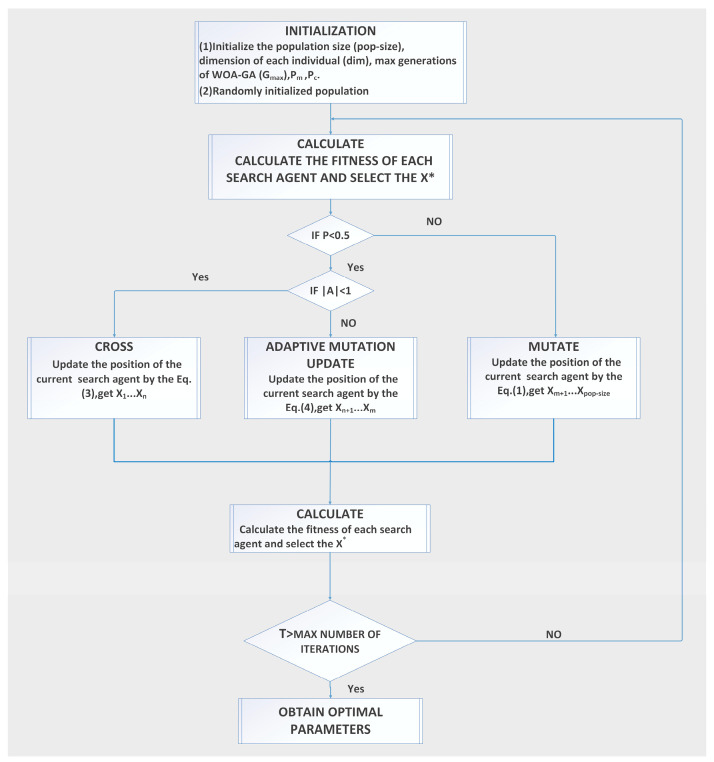
Operation diagram of WOA-GA.

Figure 4 illustrates the technical principle of the WOA-GA.

### 2.2. Modeling of Integrated Optimization

A common understanding of integration is to combine many algorithms that have different functions and are suitable for different situations to solve a complex problem. When faced with complicated tasks, a “collective intelligence” model generally performs better than a single model.

The essence of RF is ensemble learning, and the basic unit is a decision tree, which is also an effective ML method. Due to two random variables, RF is insensitive to noisy data and can avoid overfitting. The use of RF is widespread due to its good performance [32], and support vector machine (SVM) is commonly used, since it is suitable for small samples with poor information [33]. By assigning weights to the training errors in WLSSVM, the learning ability of the model is improved and noise in the training samples is effectively reduced. Therefore, in this study, hybrid models were built using WLSSVM and RF to predict the solubility of sulfur in acidic gases. Researchers should note that the performances of RF and WLSSVM are highly dependent on the choice of hyperparameters. However, thus far, there is little research on the intelligent optimization of the hyperparameters of the two model, which is usually empirically chosen. When WLSSVM or RF perform optimally in different application contexts, the hyperparameter values vary. A good ML model is difficult to obtain using selecting hyperparameters based on “empirical” criteria [17].

**Figure 5:**
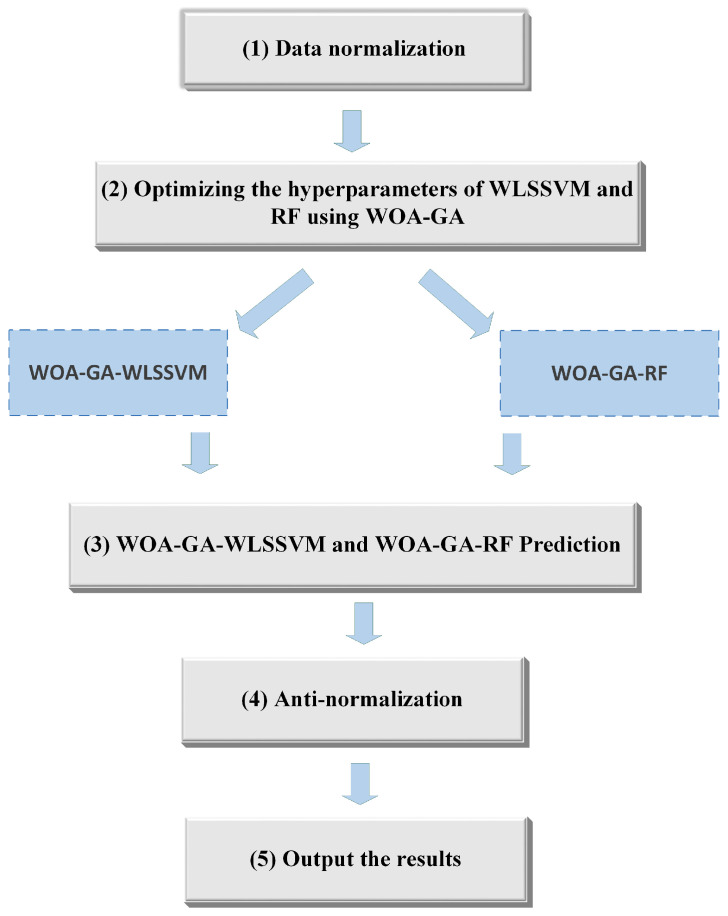
Implementation steps of the models.

As a solution to these problems, this study applies the WOA-GA in search of hyperparameters to optimize the objective function and uses the cnCV training model to obtain more information. Figure 5 shows the implementation steps of WOA-GA-WLSSVM and WOA-GA-RF.

As shown in Figure 5, the modeling process is as follows:

(1) Data preprocessing was performed first. To eliminate the effect of different units and magnitudes of variables on model training, the study normalized all data sets to between −1 and 1.
(2) The optimal hyperparameters of WLSSVM and RF were selected using WOA-GA to build the WOA-GA-WLSSVM and WOA-GA-RF models.
(3) The WOA-GA-WLSSVM and WOA-GA-RF models were trained and tested using cnCV.
(4) The data were anti-normalized.
(5) The final results were output.

### 2.3. Development of Prediction Models

#### 2.3.1. The Original Data

The prediction models were established using 281 sets of actual samples in the open study [12,16,34,35,36,37,38]. There are 55 sets of data available for pure H_2_S environments and 226 sets of data for acidic gas mixture environments. Table 3 presents a statistical summary of the data used.

To eliminate the effect of different units and magnitudes of variables on model training, the study normalized all data sets to between −1 and 1, with the following expression:

(6) $$y_{i}=\frac{2\left(x_{i}-x_{\min}\right)}{\left(x_{\max}-x_{\min}\right)}-1$$

where $y_{i}$ points out the normalized value, $x_{i}$ is the collected experimental data, $x_{\max}$ and $x_{\min}$ represent the maximum and minimum values of the data sets, respectively.

#### 2.3.2. Model Internal Parameters

The objective of the proposed prediction models is to obtain the optimum regression between sulfur solubility and multiple influencing factors. The algorithm would become more complicated and interfere with the model’s stability if all the candidate-influencing factors were included. Based on previous research findings [16], this study used temperature, pressure, H_2_S content, CO_2_ content, and CH_4_ content as input variables. Table 4 presents the main parameters.

## 3. Results

### 3.1. Comparison and Validation of Models

To determine whether the suggested model performs effectively, a rigorous evaluation is required. The average absolute relative deviation (*AARD*), root mean square error (*RMSE*), coefficient of determination (*R^2^*), standard deviation (*SD*), and correlation coefficient (*R*) are treated as assessment indicators. A global and local assessment of the models is conducted, which is calculated as follows:

(7) $$AARD=\frac{100}{N}\sum_{i=1}^{N}\left|\frac{y_{i}^{\exp}-y_{i}^{cal}}{y_{i}^{\exp}}\right|$$

(8) $$RMSE=\sqrt{\frac{1}{N}\sum_{i=1}^{N}{\left(y_{i}^{\exp}-y_{i}^{cal}\right)}^{2}}$$

(9) $$R^{2}=1-\frac{\sum_{i=1}^{N}{\left(y_{i}^{\exp}-y_{i}^{cal}\right)}^{2}}{\sum_{i=1}^{N}{\left(y_{ave}^{\exp}-y_{i}^{cal}\right)}^{2}}$$

(10) $$SD=\sqrt{\frac{1}{N-1}{\left(\frac{y_{i}^{exp}-y_{i}^{cal}}{y_{i}^{exp}}\right)}^{2}}$$

(11) $$R=\frac{\sum_{i=1}^{n}\left(y_{i}^{exp}-y_{ave}^{exp}\right)\left(y_{i}^{cal}-y_{ave}^{cal}\right)}{\sqrt{\sum_{i=1}^{n}{\left(y_{i}^{exp}-y_{ave}^{exp}\right)}^{2}\sum_{i=1}^{n}{\left(y_{i}^{cal}-y_{ave}^{cal}\right)}^{2}}}$$

where *N* is the number of samples and $y_{i}^{exp},y_{i}^{cal},y_{ave}^{exp},y_{ave}^{cal}\;$ represent the experimental values, calculated values, mean of the experimental values, and mean of the predicted values, respectively.

**Figure 6:**
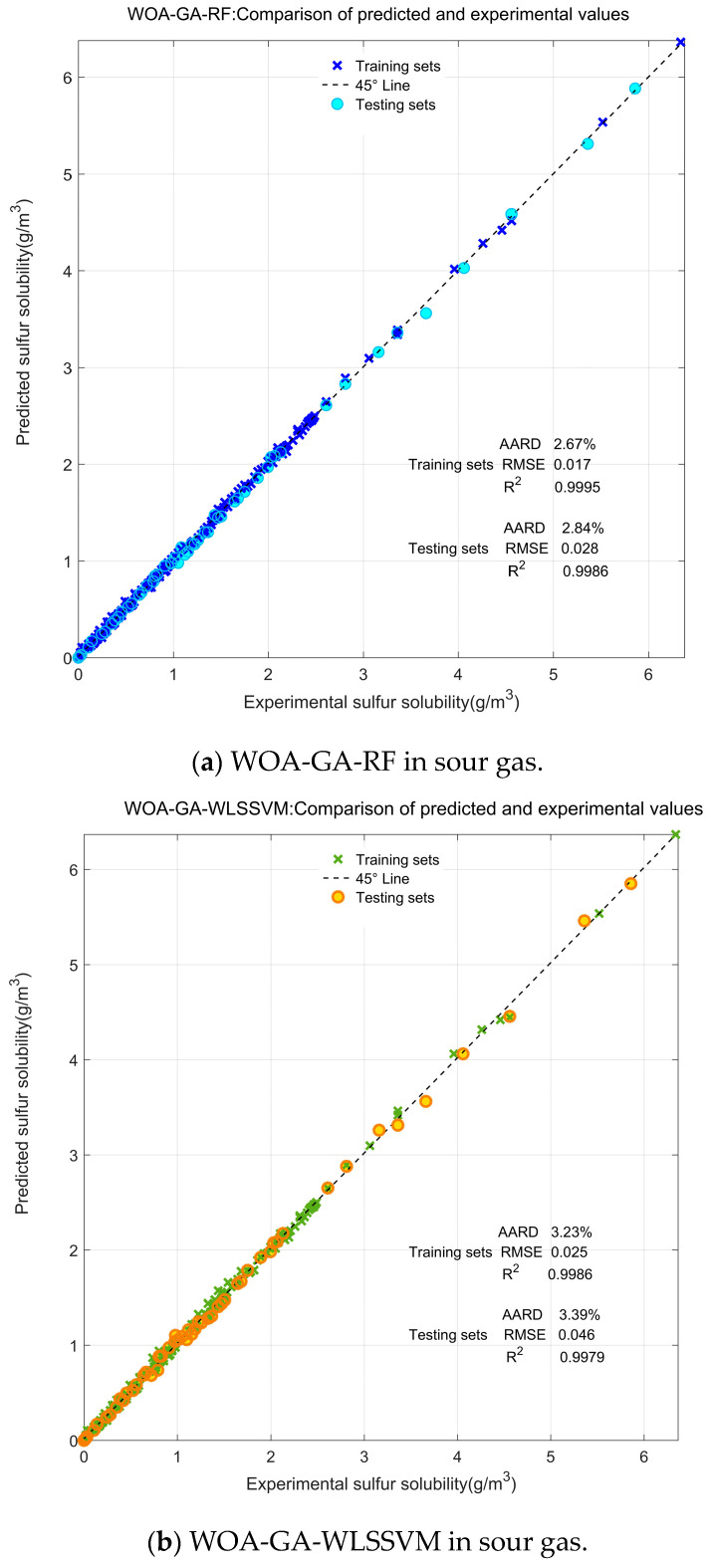

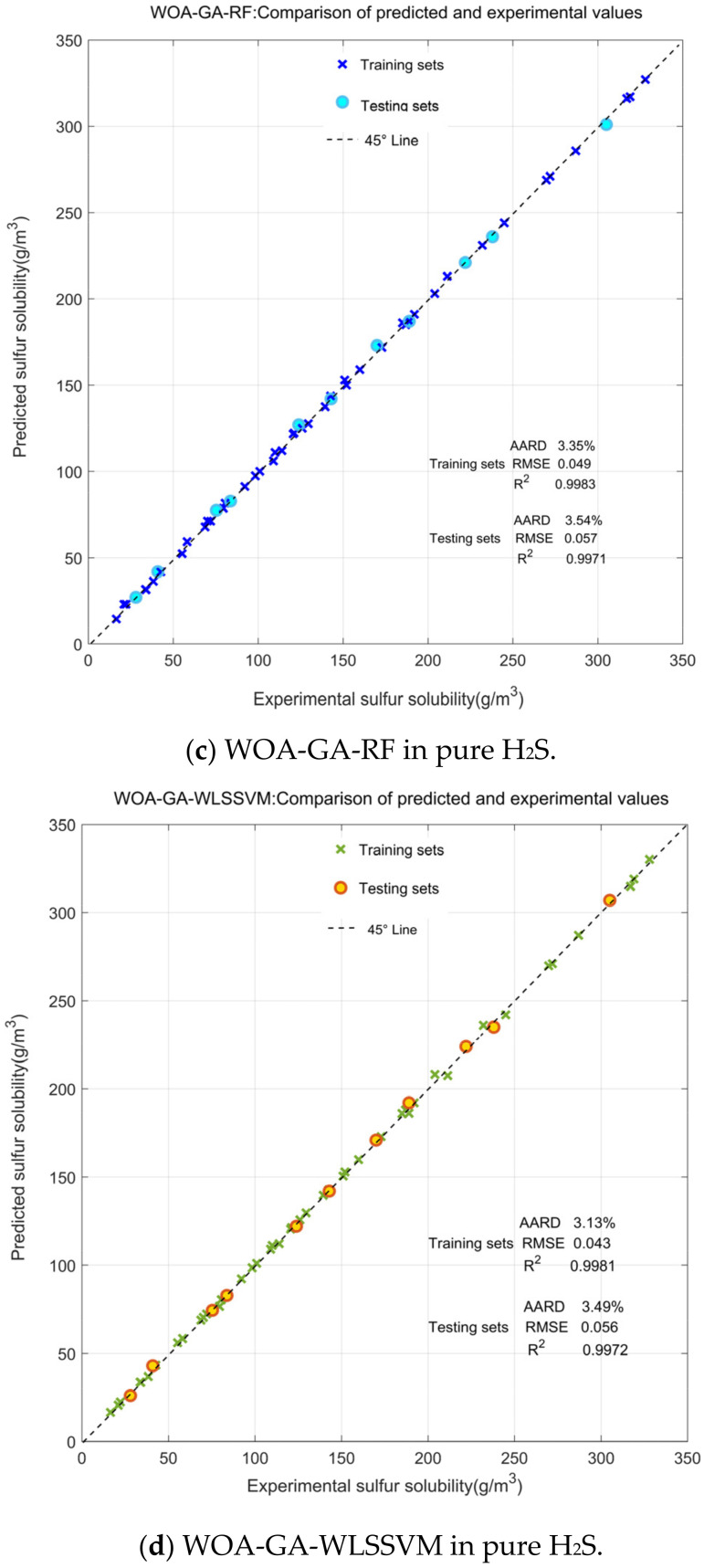
Comparison of predicted and real values of sulfur solubility: (**a**,**b**) in sour gas, (**c**,**d**) in pure H_2_S.

Figure 6 displays the optimal values of WOA-GA-RF and WOA-GA-WLSSVM during the testing phases. The reference line indicates the most ideal case, in which the predicted value exactly matches the real value. If the forecast is closer to the reference line, the forecast will be better, and vice versa. In sour gases (226 data points), the AARD and RMSE values of WOA-GA-RF on the training set are lower than those of WOA-GA-WLSSVM. The R^2^ is closer to 1 compared with that of WOA-GA-WLSSVM. This indicates that the WOA-GA-RF model has a strong fitting ability. In the testing set, the AARD values of WOA-GA-RF and WOA-GA-WLSSVM are 2.84% and 3.39%, respectively. The *R^2^* is 0.9986 and 0.9979, respectively. According to this, the calculated results of both models are very close to reality. In comparison, WOA-GA-RF has a higher accuracy and better precision. In pure H_2_S (55 data points), the values of both AARD and RMSE for WOA-GA-WLSSVM are slightly lower than those of WOA-GA-RF. This indicates that WOA-GA-WLSSVM outperforms WOA-GA-RF by a slight margin. Compared to the sour gas background (larger sample size), this result is different. WOA-GA-WLSSVM outperforms WOA-GA-RF in sour gases, whereas WOA-GA-WLSSVM outperforms WOA-GA-RF in pure H_2_S. This phenomenon is likely caused by the difference in sample size (the sample size on a pure H_2_S background is smaller than on a sour gas background). Since WLSSVM is a support vector-based model, the sample size may have less of an impact on the model’s results.

**Figure 7:**
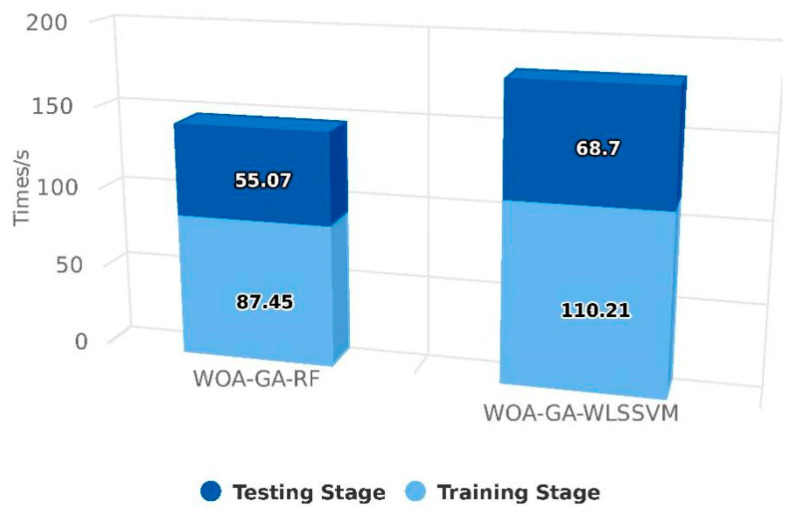
Model running time comparison.

Figure 7 shows the time spent by the two models in different training and testing stages. The training time of WOA-GA-WLSSVM is longer, and the overall time is 36.39 s longer than that of WOA-GA-RF. With the growth in data volume, the advantages of RF become more significant, whereas WLSSVM encounters significant computational bottlenecks. Because of the low time complexity of RF, the model training speed is relatively fast, especially for large datasets. The time complexity of WLSSVM is O(n^2^) (where n is the size of the training set), which may increase the time and reduce the efficiency of the model when applied to large datasets [39]. As a result, WOA-GA-RF has proven to be more effective at predicting sulfur solubility and is recommended in this study.

**Figure 8:**
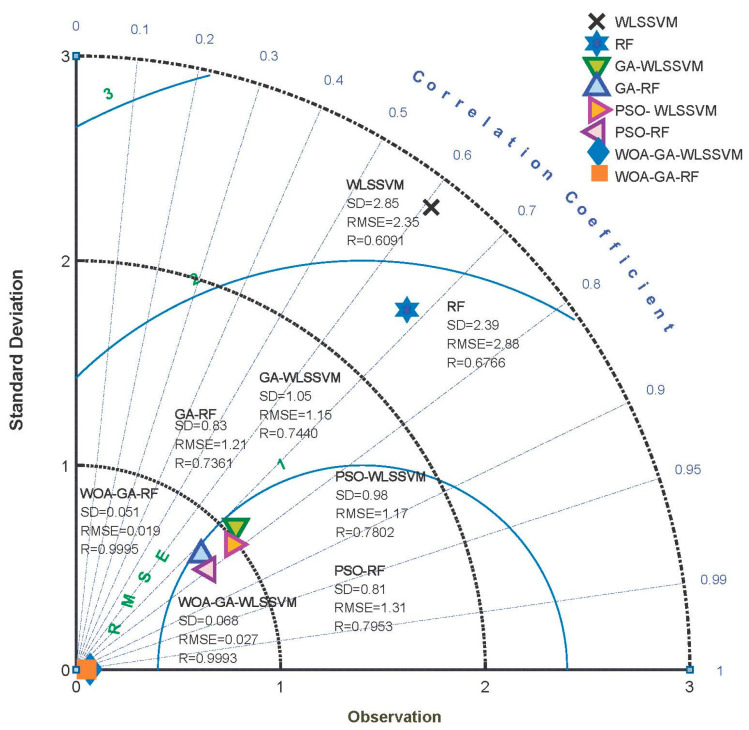
Comparison of similar models.

The study tested the improvement effect of WOA-GA on the models (on all datasets) using Taylor diagrams, comparing WOA-GA-WLSSVM, WOA-GA-RF, GA-WLSSVM, GA-RF, PSO-WLSSVM, PSO-RF, RF, and WLSSVM. This method presents three-dimensional data on a two-dimensional plane to provide a comprehensive and clear picture of the model’s performance in various dimensions. In the Taylor diagram, scatter points represent the model, radial lines represent R, horizontal and vertical axes represent SD, and dashed lines represent RMSE. As shown in Figure 8, the values of SD for WLSSVM and RF are 2.85 and 2.39, respectively, and the values of RMSE are 2.35 and 2.88, respectively. The error is much higher than the other six models that have been optimized by the metaheuristic algorithm, and the prediction accuracy is the lowest. The R values, which are 0.6091 and 0.6776, respectively, are low compared to other similar models, indicating that the fitting ability is also unsatisfactory. With the optimization of GA and PSO, the R of GA-WLSSVM and GA-RF improved slightly. As a result of the shortcomings of the premature convergence and poor convergence performance of GA and PSO, large prediction errors remain for GA-WLSSVM, GA-RF, PSO-WLSSVM, and PSO-RF. The predicted value is not well matched with the true value. The values for SD, RMSE, and R of WOA-GA-RF are 0.051, 0.019, and 0.9995, respectively. In WOA-GA-WLSSVM, SD, RMSE, and R are 0.068, 0.027, and 0.9993, respectively. Compared with the remaining six similar models, WOA-GA-RF and WOA-GA-WLSSVM provide a good accuracy and fit capability. Thus, WOA-GA proves to be an effective method of optimizing WLSSVM and RF, as well as improving model performance.

Table 5 compares the proposed model with commonly used empirical models and ML models to further verify its performance [14,16,34,36,40,41]. This study found that, compared to empirical models, ML methods are more effective, providing a closer match to the experimental data, indicating that they are reliable and relevant. In terms of both AARD and RMSE, the ML models outperformed the empirical models. The ML model developed by Bian is also of the SVM type. Nevertheless, the performance of the model is not as effective as WOA-GA-WLSSVM in each index, which may suggest that the hyperparameter search is an important factor in improving model performance. Among all models, the WOA-GA-RF model reached the minimum value with an AARD of 2.69% and RMSE of 0.019, respectively. This is not very different from the R^2^ of the ML model, which is almost always above 0.99. In summary, the WOA-GA-RF model provides the best overall performance and best represents the actual sulfur solubility variation pattern observed in the current study. The results described in Section 3.1 show that accurate values and patterns of variations in sulfur solubility (within a certain range) can be efficiently obtained. Moreover, the proposed model outperforms current machine learning methods in terms of accuracy in predicting sulfur solubility.

### 3.2. Stability Analysis

The stability of a machine learning model is fundamentally different from its performance, and researchers cannot simply identify this stability by assessing its accuracy. There is a direct correlation between the stability of the model and its effectiveness in practical applications.

In this study, cross-validation correctness was used as an indicator of model stability. The dataset was divided into five parts, four of which are used, in turn, for training, and one of which is used for testing. Table 6 shows the correctness of the two ML methods over the five tests. The correctness for both models is above 0.9, indicating that the model has good performance. It was found that the correctness of WOA-GA-WLSSVM varies between 0.9011 and 0.9857, and even though the mean correctness is as high as 0.9467, the SD is nearly twice the variance of the WOA-GA-RF, which indicates that the WOA-GA-WLSSVM’s performance is not particularly stable across the different test sets. Because RF is considered the beginning of ensemble tree models, and multiple decision trees are used to make decisions together, the model stability may be higher than WLSSVM. As a matter of course, the prediction result depends on the selection of test data, as well as on the number of tests conducted.

### 3.3. Reliability Analysis

Reliability analysis is usually carried out using the leverage method [42]. Based on the Williams plot, a model is statistically reliable if the majority of data points are clustered within a square. Detailed definitions can be found in the literature [43].

**Figure 9:**
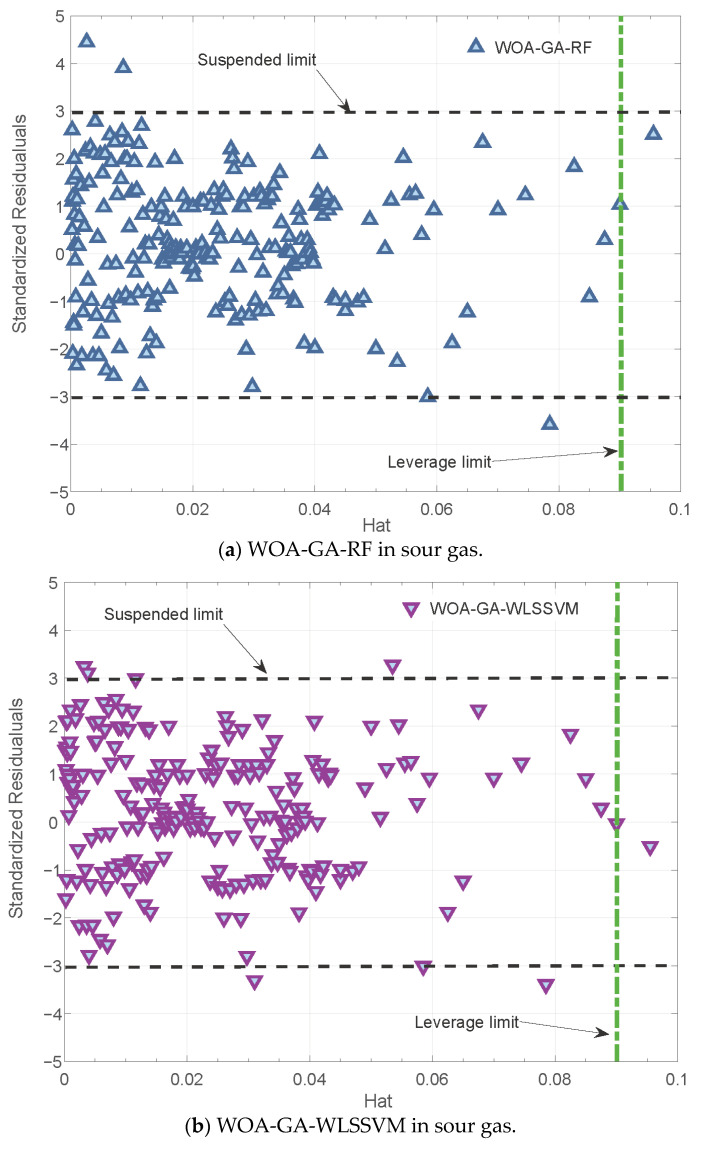

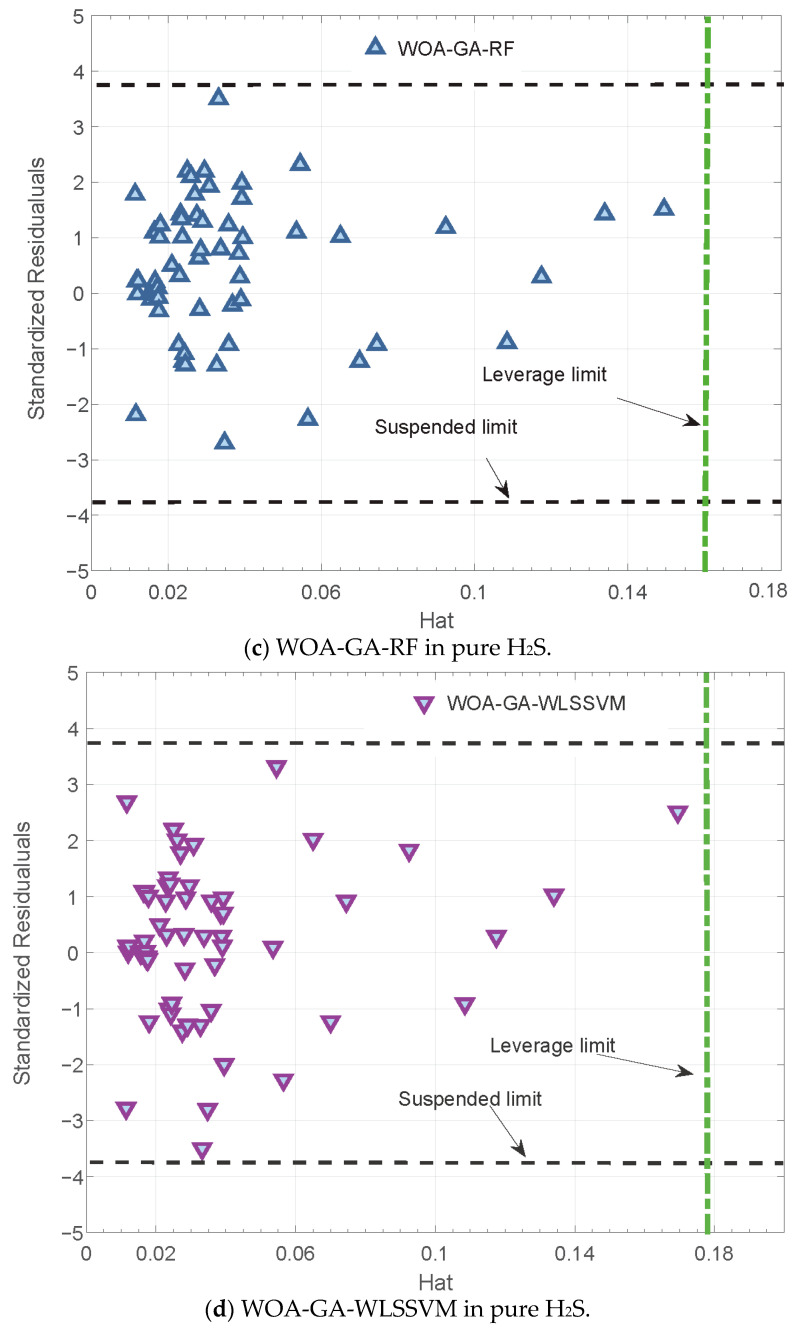
Diagnosis of abnormal data.

Figure 9 shows the Williams plots after outlier detection from both new models. Both the WOA-GA-RF and WOA-GA-WLSSVM have only isolated abnormal data, which indicates that both models passed the statistical test.

### 3.4. Analysis of the Contribution of Features

**Figure 10:**
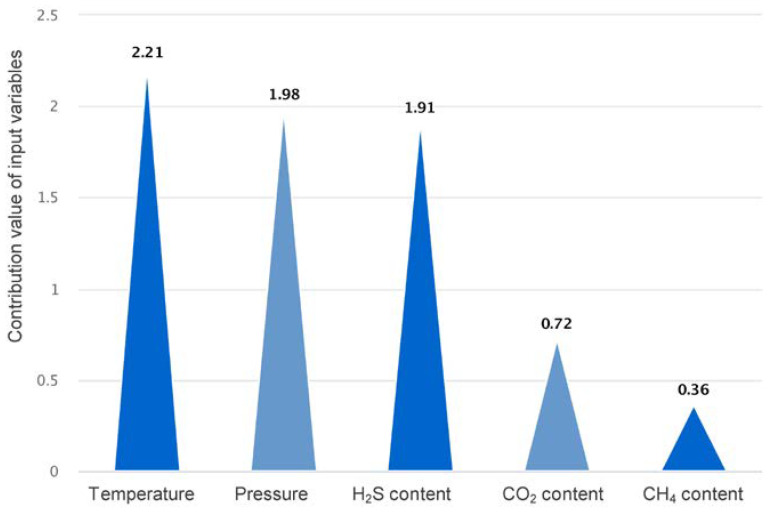
The contribution of input variables.

In addition to its predictive accuracy, the interpretability of ML models is of equal importance. Compared to commonly used measures, such as Pearson (PR) correlation, gPOIM is able to avoid exaggerating the contribution of input variables and accurately assess the effect of the non-monotonicity of input variables; it is now used in practical engineering applications [44,45]. To compensate for the black box nature of the ML model, the study used gPOIM to visualize the contribution of the input variables, as shown in Figure 10. The order of importance of the input variables to sulfur solubility is T > P > H_2_S > CO_2_ > CH_4_. Temperature (T) has absolute control on sulfur solubility, and pressure (P) and H_2_S content also play a significant role. The study of Bian et al. [13] concluded that pressure was the most important factor, whereas CO_2_’s effect on solubility can be ignored. This is most likely caused by the error in the PR when analyzing the non-linear relationship between pressure, CO_2_, and solubility [46].

**Figure 11:**
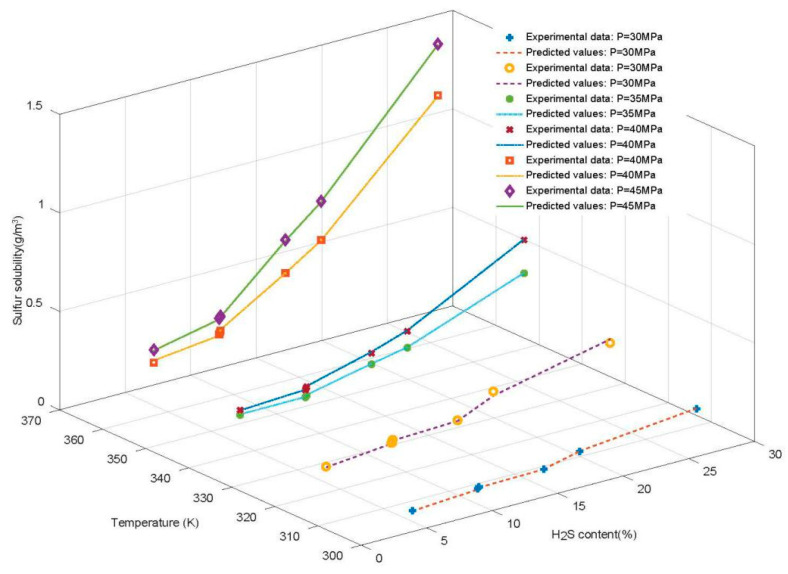
Comparison under various conditions.

In Figure 11, actual and predicted values for various temperature, pressure, and H_2_S content conditions are shown (taking WOA-GA-RF as an example). The predicted curve is highly consistent with the experimental value, and temperature, pressure, and sulfur content all contribute to the solubility of sulfur. The sulfur solubility significantly increases when the H_2_S content exceeds 10%, with other conditions (temperature and pressure) remaining equal. For example, at a pressure of 45 MPa and a temperature of 363.2 K, sulfur solubility is 0.284 g/m^3^ when the H_2_S content is 4.95%, 0.366 g/m^3^ when the H_2_S content is 10.03%, and 0.666 g/m^3^ when the H_2_S content is 14.98%. There is a clear cut-off point of 10% H_2_S content. With the increase in H_2_S content, sulfur solubility rapidly grows after this cut-off point. A rise in temperature and pressure also significantly increased sulfur solubility. Under the conditions of 40 MPa pressure and 14.98% H_2_S content, the sulfur solubility values are 0.287 g/m^3^ and 0.497 g/m^3^ at temperatures of 343.2 K and 363.2 K, respectively. Under the conditions of a 363.2 K temperature and 14.98% H_2_S content, the sulfur solubility values are 0.497 g/m^3^ and 0.666 g/m^3^ at pressures of 40 Mpa and 45 Mpa, respectively.

The solubility pattern of sulfur is shown in Figure 11. High temperature and pressure promote the solubility of sulfur, and the higher the H_2_S content, the more pronounced this promoting effect. Operators can manipulate the dissolution of sulfur by adjusting the temperature and pressure levels during the transportation and processing of natural gas containing sulfur. Furthermore, sulfur solubility studies may serve as a basis for or provide new perspectives in a variety of investigations. For example, researchers can choose an environmentally friendly sulfur solvent that is more soluble in sulfur and easier to separate and recover when purifying natural gas containing sulfur, depending on sulfur dissolution patterns.

## 4. Conclusions

To help researchers obtain accurate information on sulfur solubility and sulfur dissolution patterns, two integrated optimization ML models were developed to predict the solubility of sulfur in sour natural gas. The main findings of this study can be summarized in the following three points.

(1) In addition to improving the diversity of algorithms, WOA-GA also optimizes the performance of traditional WLSSVM and RF models while avoiding their original drawbacks. By incorporating cnCV in modeling, limited data can provide sufficient information to effectively train the ML model. Researchers should carefully consider the trade-off between computational precision and cost and select ML methods according to the task context, minimizing research costs while ensuring goal completion.
(2) RF was used to predict sulfur solubility for the first time, and its accuracy, stability, and reliability were verified. Compared to the existing ML model, the WOA-GA-RF model has a better comprehensive performance and a greater prediction accuracy in sulfur solubility, with an AARD of 2.69%, SD of 0.051, RMSE of 0.019, and R^2^ of 0.9991.
(3) Sulfur solubility was found to be more affected by temperature, pressure, and H_2_S content. Temperature is the most significant element influencing sulfur solubility, followed by pressure. Sulfur solubility significantly increases when the H_2_S content exceeds 10%, and other conditions remain the same. This pattern can be used to set the relevant parameters in the processing of natural gas containing sulfur.

It should be noted that the amount of sulfur solubility data used in the study is small, and the hybrid models are computationally efficient. In the future, more research is needed to verify the effectiveness and operability of the models when faced with complex tasks and larger sample sizes. It is possible to extrapolate the research conclusions to temperatures and pressures that are higher than the experimental data ranges, but these need to be verified before being applied to real-life situations.

## Figures and Tables

**Table 1:** ML models for obtaining sulfur solubility.

Authors	Models	Category	Number of Data	Scope of Data	Input Dimension	Results	Possible Disadvantages
Chen L (2014) [15]	GA-LM-BP	ANN	74	303.20–363.20 K 11.82–40 Mpa	5	AARD = 5.54%	Inefficient and irregular coding of GA leads to inaccurate results
Chen HS (2019) [14]	CFA-SVR	SVM	110	316.26–433.15 K 6.89–60 Mpa	5	AARD = 4.24% RMSE = 0.0401	The late convergence speed of CFA is slow and easily falls into the local optimum
Bian XQ (2020) [13]	GWO-LSSVM	SVM	239	303.20–433.15 K 10–60 Mpa	5	AARD = 3.50% RMSE = 1.0832	GWO easily falls into the local optimum, and the convergence accuracy is not high
Fu L (2020) [16]	T-S FNN	ANN	167	303.15–433.15 K 10–66.52 Mpa	5	AARD = 5.35% RMSE = 0.0600	T S-FNN is slower to learn, prone to local minima, and may not even function properly
Amar MN (2020) [9]	CFNN	ANN	239	303.20–433.15 K 7.03–60 Mpa	5	RMSE = 0.0488	The learning speed of CFNN is slow, and the ability to obtain a global optimal solution is weak

Artificial neural network (ANN), support vector machine (SVM), genetic algorithm (GA), chaos-based firefly algorithm (CFA), grey wolf optimizer (GWO), T-S fuzzy neural network (T-S FNN), cascaded forward neural network (CFNN), average absolute relative deviation (AARD), root mean square error (RMSE).

**Table 2:** The disadvantages of k-fold CV and nCV.

Disadvantages of k-fold cross-validation (k-fold CV)	Disadvantages of nested cross-validation (nCV)
a. Overly optimistic results of the assessment b. Data characteristics cannot be fully learned c. Knowledge leakage	a. Excessive calculation b. Complicates the model c. Selects irrelevant features

**Table 3:** Statistical summary of the sulfur solubility data used.

	Symbol	Unit	Min	Max
Temperature	T	K	303.2	433.15
Pressure	P	Mpa	7	66.52
H_2_S content	XH_2_S	%	2.93	100

**Table 4:** The model’s detailed composition.

Parameter	Value
Input data form	[−1, +1]
Input variables	5
Max iterations	200
Population	30
Encoding length	7
Crossover probability *P_c_*	0.7
Mutation probability *P_m_*	0.3
Kernel function	Gaussian radial basis (RBF)
Penalty parameter	2.1089
Kernel function parameter	12.5165

**Table 5:** Comparison of common models.

Models	*AARD* (%)	*SD*	*RMSE*	*R* ^2^
Roberts model (empirical model)	65.36	0.86	0.67	0.6792
Guo-Wang model (empirical model)	12.84	0.15	0.17	0.9833
Hu model (empirical model)	17.32	0.22	0.21	0.9731
Fu L model (T-S FNN)	5.35	0.08	0.06	0.9983
Bian XQ model (GWO-LSSVM)	3.50	0.08	0.024	0.9976
Chen HS model (CFA-SVR)	4.24	0.07	0.04	0.9978
WOA-GA-WLSSVM	3.33	0.068	0.027	0.9988
WOA-GA-RF	2.69	0.051	0.019	0.9991

**Table 6:** Analysis of correctness.

Number	WOA-GA-WLSSVM	WOA-GA-RF
1	0.9011	0.9302
2	0.9651	0.9291
3	0.9016	0.9301
4	0.9857	0.9681
5	0.9801	0.9697
Mean Correctness	0.9467	0.9454
Standard Deviation	0.0377	0.0192

## Data Availability

Data of this study are available on request from the corresponding author.
